# Genetic diversity and population structure of early and extra-early maturing maize germplasm adapted to sub-Saharan Africa

**DOI:** 10.1186/s12870-021-02829-6

**Published:** 2021-02-17

**Authors:** Baffour Badu-Apraku, Ana Luísa Garcia-Oliveira, César Daniel Petroli, Sarah Hearne, Samuel Adeyemi Adewale, Melaku Gedil

**Affiliations:** 1 International Institute of Tropical Agriculture (IITA), PMB 5320, Oyo Rd, Ibadan, 200001 Nigeria; 2 International Maize and Wheat Improvement Center (CIMMYT), Carretera México-Veracruz Km. 45 El Batán, 56237 Texcoco, Mexico

**Keywords:** Maize (*Zea mays* L.), Genetic diversity, Population structure, DArT markers, Heterotic grouping

## Abstract

**Background:**

Assessment and effective utilization of genetic diversity in breeding programs is crucial for sustainable genetic improvement and rapid adaptation to changing breeding objectives. During the past two decades, the commercialization of the early and extra-early maturing cultivars has contributed to rapid expansion of maize into different agro-ecologies of sub-Saharan Africa (SSA) where maize has become an important component of the agricultural economy and played a vital role in food and nutritional security. The present study aimed at understanding the population structure and genetic variability among 439 early and extra-early maize inbred lines developed from three narrow-based and twenty-seven broad-based populations by the International Iinstitute of Tropical Agriculture Maize Improvement Program (IITA-MIP). These inbreds were genotyped using 9642 DArTseq-based single nucleotide polymorphism (SNP) markers distributed uniformly throughout the maize genome.

**Results:**

About 40.8% SNP markers were found highly informative and exhibited polymorphic information content (PIC) greater than 0.25. The minor allele frequency and PIC ranged from 0.015 to 0.500 and 0.029 to 0.375, respectively. The STRUCTURE, neighbour-joining phylogenetic tree and principal coordinate analysis (PCoA) grouped the inbred lines into four major classes generally consistent with the selection history, ancestry and kernel colour of the inbreds but indicated a complex pattern of the genetic structure. The pattern of grouping of the lines based on the STRUCTURE analysis was in concordance with the results of the PCoA and suggested greater number of sub-populations (K = 10). Generally, the classification of the inbred lines into heterotic groups based on SNP markers was reasonably reliable and in agreement with defined heterotic groups of previously identified testers based on combining ability studies.

**Conclusions:**

Complete understanding of potential heterotic groups would be difficult to portray by depending solely on molecular markers. Therefore, planned crosses involving representative testers from opposing heterotic groups would be required to refine the existing heterotic groups. It is anticipated that the present set of inbreds could contribute new beneficial alleles for population improvement, development of hybrids and lines with potential to strengthen future breeding programs. Results of this study would help breeders in formulating breeding strategies for genetic enhancement and sustainable maize production in SSA.

**Supplementary Information:**

The online version contains supplementary material available at 10.1186/s12870-021-02829-6.

## Background

During the twentieth century, the advances in plant science, especially genetics in conjunction with statistics, have enhanced the progress in the selection of agronomically desirable genotypes following systematic reshuffling of the genome in crop plants including staple food crops through breeding. This has resulted in unprecedented improvement in food production which is expected to continue to play a vital role in the world food security [1, 2]. Even though these breeding efforts have fulfilled the demands of intensive agriculture, it has been postulated that selective breeding may lead to the narrowing of the genetic base of crop plants which could seriously jeopardize future crop improvement efforts [3].

Following the green revolution which has benefited mainly continents that host developing countries, there has been an increase in awareness regarding the importance of genetic diversity in food crops [4, 5]. During the past 5 decades, the Consultative Group for International Agricultural Research (CGIAR) breeders have been actively contributing to the broadening of the genetic bases of their mandate crops worldwide, especially in the third world, through provision of elite genetic materials to their national partners [6, 7]. It has been a routine practice for breeders to infuse new genetic diversity into their base populations depending on the breeding objectives [8]. However, this effort has not resulted in marked changes in the diversity of field crops including major cereals such as maize, rice and wheat [3].

Of the cereal food crops, maize is perhaps the most important for food and economic security in SSA including in West and Central Africa (WCA), covering about a quarter of the total land area under cereal production in the sub-region [9–11]. However, in this sub-region maize is considered as a multipurpose crop, which is consumed predominantly as a staple food crop by humans as well as poultry feed and raw material for livestock industries [12, 13]. In an effort to promote the production of the early and extra-early maize varieties in SSA particularly in WCA, IITA collaborated with the International Maize and Wheat Improvement Center (CIMMYT) and the National Agricultural Research Institutes (NARIs) of WCA in 1987 to initiate systematic research efforts to develop source populations combining earliness with tolerance to moisture stress under the Maize Research Network (WECAMAN) [14]. Since then, other beneficial traits such as resistance/tolerance to maize streak virus (MSV), parasitism to *Striga*, low N and enhanced nutritional quality (such as quality protein and pro-vitamin A) have also been introgressed into the early and extra-early maize by the IITA-MIP [15].

In maize breeding, germplasm from similar heterotic groups and with desirable agronomic characteristics are usually intermated. Consequently, genotypes of different heterotic groups are separately kept to ensure that the developed populations are heterotic. Through this strategy, inbreds generated from different populations are normally heterotic when crossed, thus giving rise to productive hybrids. For example, in a cross between populations A and B, if the resulting F_1_ performed better than the mean of the two parental populations, then the F_1_ is described as exhibiting mid-parent heterosis. In contrast, if the performance of the F_1_ is superior to that of the better parent, it is described as exhibiting better parent heterosis. In either case, the breeder is guaranteed progress from selection for genetic enhancement of the trait of interest. Derived inbred lines from narrow and/or broad-based populations should also display heterosis as an evidence of high specific combining ability. Such inbred lines are useful as parental lines for commercial hybrid development. These concepts have been used extensively in the IITA-MIP program to develop three narrow-based and twenty-seven broad-based source populations which have been taken through several cycles of improvement followed by the extraction of several multiple-stress tolerant inbred lines for hybrid development. These inbred lines exhibit contrasting degrees of resistance and/or tolerance to *S. hermonthica*, low N as well as drought stress. Some of the lines are parents of hybrids released in different countries, in SSA in different agro-ecological zones [16].

Classifying inbred lines into heterotic groups is important for exploiting their potential worth in the development of outstanding hybrids and synthetics as well as for developing new heterotic groups. It is therefore of utmost importance to study the extent of genetic variability and heterotic groups in the early and extra-early inbred lines in the IITA-MIP. Information on the genetic diversity and heterotic groups in the early and extra-early inbreds would be beneficial to the hybrid program at IITA as well as the national maize programs in SSA.

During the past two decades, the integration of molecular markers into the IITA-MIP has further facilitated the improvement of the efficiency of the breeding process, resulting in rapid generation of multiple stress tolerant early and extra-early maturing maize varieties and hybrids with enhanced nutritional quality for the countries of WCA [16, 17]. This is partly due to the low cost and efficiency of molecular makers as a result of the remarkable technological advancement in molecular genetics, resulting in improvement of DNA-based markers over biochemical and morphological markers. In addition to the cost efficiency, other advantages of DNA markers such as abundance and even distribution throughout the genome, relatively rapid and efficient detection, lower genotyping error rates and generally neutral effect of allelic variation on individuals have made them ideal candidates for utilization in breeding processes [18]. The application of molecular markers for characterization of inbred lines complements and perfects classification into heterotic groups based on combining ability [19]. SNP markers are widely distributed and the most abundant molecular markers throughout the genomes of crop plants, thus making them the most commonly used in genetic studies [20]. The Diversity Arrays Technology (DArT) in combination with the next-generation sequencing platforms known as DArTseq™ [21–23] has been recently introduced. This has provided a good alternative of high throughput marker genotyping platform, and due to its nature, is a perfect option for diversity analysis. The DArTseq has several advantages prominent among which are no prior knowledge about sequencing of the plant genome and the capacity to produce high-density results, possibility to score thousands of unique genomic-wide DNA fragments in a single experiment with low-cost genotype information [24, 25]. The DArTseq method has been used in discriminating different species for population studies, diversity studies, characterization of germplasm and studies involving genome-wide association [26–28].

Information on diversity is important for estimating the amount of genetic diversity lost due to conservation or selection [29, 30]. Acquaah [31] pointed out that the diversity and relatedness among inbred lines obtained from the same population or different populations are necessary in deciding the best breeding strategies to be employed to maximize their potential in a breeding program. Furthermore, combination of pedigree information and genetic distance estimates could be invaluable for placing inbred lines in distinct heterotic groups to help prevent crosses between closely related lines [32]. In order to design the most appropriate product development strategies for successful harnessing of heterosis in maize, comprehension of the extent and patterns of diversity and the relationship among the base materials is crucial for developing new inbred lines, and the choice of testers for selecting outstanding inbred line combinations for hybrid development programs [33].

Towards this end, several studies have been carried out at the molecular level to determine the diversity in the IITA-MIP inbreds, including the early and extra-early inbred lines, but these studies were conducted mostly with either few molecular markers or a limited number of inbred lines developed at specific periods in the IITA-MIP [34–36]. Thus, there is a need to assess the genetic differences and inter-relationships among the old and new early and extra-early maturing white, orange and yellow endosperm maize inbreds extracted by the IITA-MIP for effective placement into heterotic groups as well as facilitate successful parent selection for hybrid development.

For the purpose of comprehensive and systematic characterization of the early and extra-early maize inbreds developed in IITA-MIP, 439 early and extra-early inbred lines including some widely used inbred lines by national maize breeders of the savanna agro-ecological zones of WCA, standard testers and parents of some early and extra-maturing hybrids released for cultivation in Nigeria, Ghana and Mali were assembled for this study. These inbred lines were developed in different breeding eras during the past three decades by introgressing novel traits from landraces and exotic germplasm sources including wild relatives such as *Zea diploperennis*. The present study assessed the genetic diversity and population structure of these inbreds using 9642 DArTseq SNP markers.

## Results

### Summary statistics of SNP markers and diversity analysis

Among the 18,927 SNPs utilized for the DArTseq genotyping of the inbreds in the present study, 12,485 SNP markers with call rate > 0.8 were informative. Thereafter, markers with minor allele frequency < 0.05 and monomorphic markers were eliminated, resulting in 9642 high quality informative SNPs which were used for further analysis. Of these markers, a total of 1370, 1123, 987, 951, 1047, 710, 734, 793, 706 and 622 SNPs were mapped on chromosomes 1 to 10, respectively. Diversity indices statistics across the 9642 SNPs indicated an average minor allele frequency (MAF) of 0.173 and polymorphic information content (PIC) of 0.206 with a range of 0.015 to 0.500 and 0.029 to 0.375, respectively (Table 1). The mean expected heterozygosity (0.249) was higher than the observed heterozygosity (0.059) values. Of the 9642 SNP markers, 3930 (40.8%) markers showed PIC values greater than 0.25 and were found to be highly informative.

**Table 1:** Diversity indices statistics of 439 early and extra-early maize inbred lines based on 9642 SNP markers

	Minor allele frequency (MAF)	Heterozygosity expected (He)	Heterozygosity observed (Ho)	Polymorphic information content (PIC)
Minimum	0.015	0.030	0.000	0.029
Median	0.132	0.229	0.051	0.203
Maximum	0.500	0.500	0.199	0.375
Mean	0.173	0.249	0.059	0.206

**Fig. 1:**
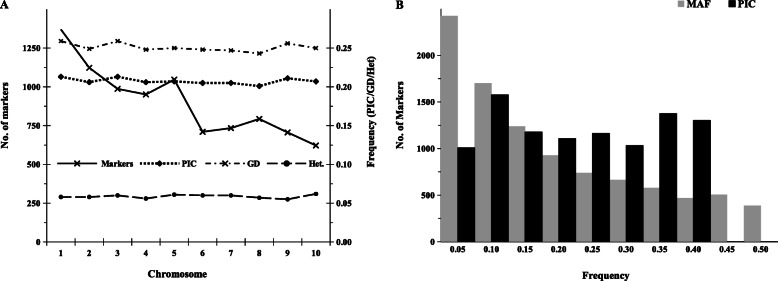
Summary statistics of 9642 DArT markers used for genotyping of 439 inbred lines: (**a**) Number of markers, mean polymorphism information content (PIC), gene diversity distribution and heterozygosity across chromosomes and (**b**) distribution of DArT markers among different minor allele frequency (MAF) and PIC value classes

The analysis of chromosome-wise informative SNP markers revealed that SNP markers varied from 622 on chromosome 10 to 1370 on chromosome 1 with an average of 904 markers per chromosome. The gene diversity (GD), PIC and heterozygosity values among chromosomes were consistent and displayed slight variations among chromosomes. The observed GD among the inbred lines varied from 0.243 on chromosome 8 to 0.259 on chromosomes 1 and 3, PIC varied from 0.201 on chromosome 8 to 0.213 on chromosomes 1 and 3 and heterozygosity ranged from 0.055 on chromosome 9 to 0.062 on chromosome 10 (Fig. 1a). PIC was uniformly distributed among the SNPs with values varying from 0.1 to 0.4, but the distribution of MAF values was asymmetrical and skewed towards lower values. More than two-fifth of the markers (42.8%) had a MAF value in the range of 0.01 to 0.10 (Fig. 1b).

### Population structure analysis

**Fig. 2:**
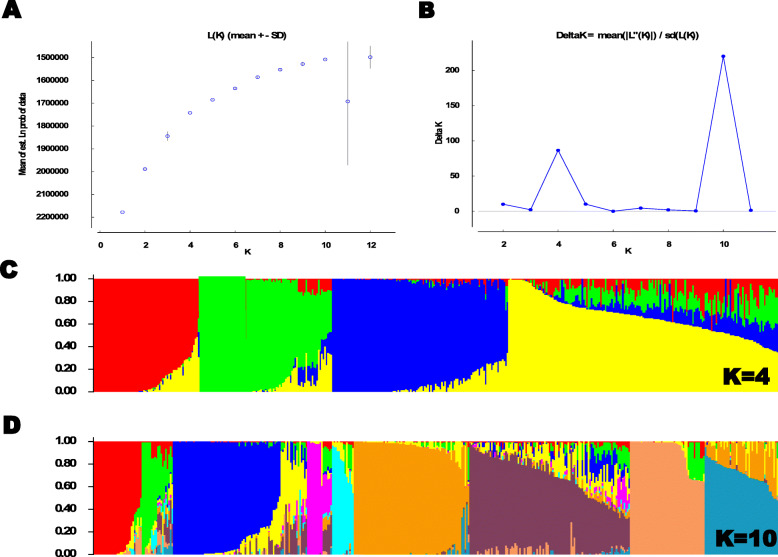
Genetic structure of the 439 early and extra-early maize inbred lines estimated with 9642 DArT markers: (**a**) The number of sub-populations determined by the LnP(D), (**b**) K model with an elevated ΔK values calculated for K varying from 1 to 12.; (**c**) and (**d**) Population structure analysis of the 439 early and extra-early inbred lines at K = 4 and 10, respectively

The different complementary approaches such as STRUCTURE, Neighbour-Joining phylogenetic trees and PCoA were employed to obtain the information on the population structure of the panel of inbred lines. The value of LnP(D) increased continuously from K = 1 to K = 12; nonetheless, an inflexion point was observed before K = 4 that was obvious after K = 10 (Fig. 2a). The highest *K* model with an elevated ΔK (K = 10), but K *=* 4 also had high ΔK values (Fig. 2b). Based on the admixture model in the software STRUCTURE at K = 4 and K = 10, the maize inbred panel of 439 inbred lines was grouped into four and ten sub-populations, respectively, using 9642 SNP markers (Fig. 2c and d). Introducing different assignment thresholds (0.9, 0.8, 0.7 or 0.6) resulted in greater decrease in the number of unassigned inbred lines (Additional file 1: Figure S1). Nonetheless, 13.1 and 15.5% of the inbred lines in the panel showed probability of association less than 60% and were considered as admixture at K = 4 and K = 10, respectively. Of these admixture lines in the panel, 31 inbreds were found to be common at both K = 4 and K = 10 (Additional file 2: Table S1).

**Fig. 3:**
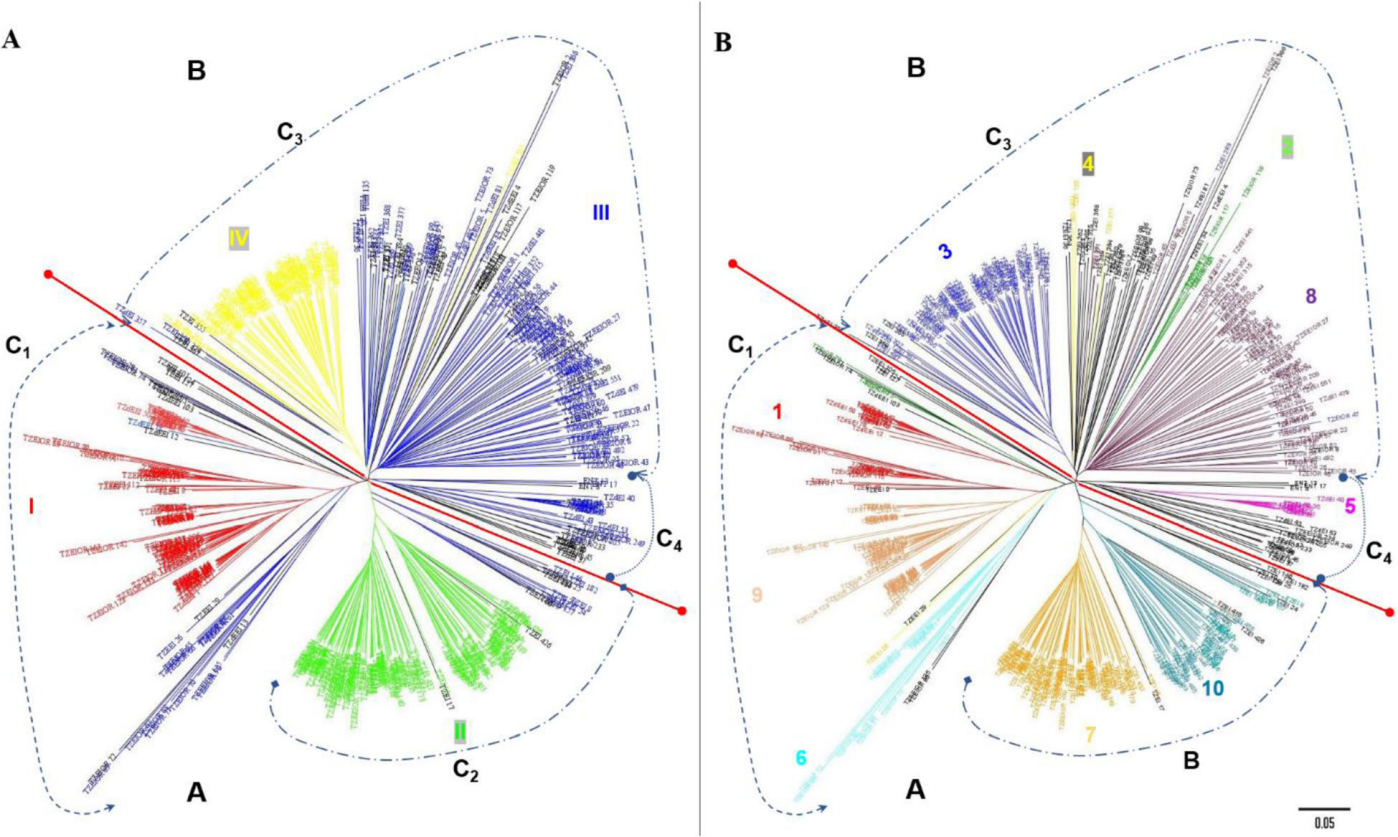
Neighbor-joining phylogenetic trees of the 439 maize inbred lines based on 9642 DArT markers. NJ trees compared with STRUCTURE results A) K = 4 and B) K = 10. The colour patterns are equivalent to the STRUCTURE analysis where individuals were assigned to their respective sub-populations/groups based on a 60% of threshold cutting. Black colour represents admixture inbred lines

**Fig. 4:**
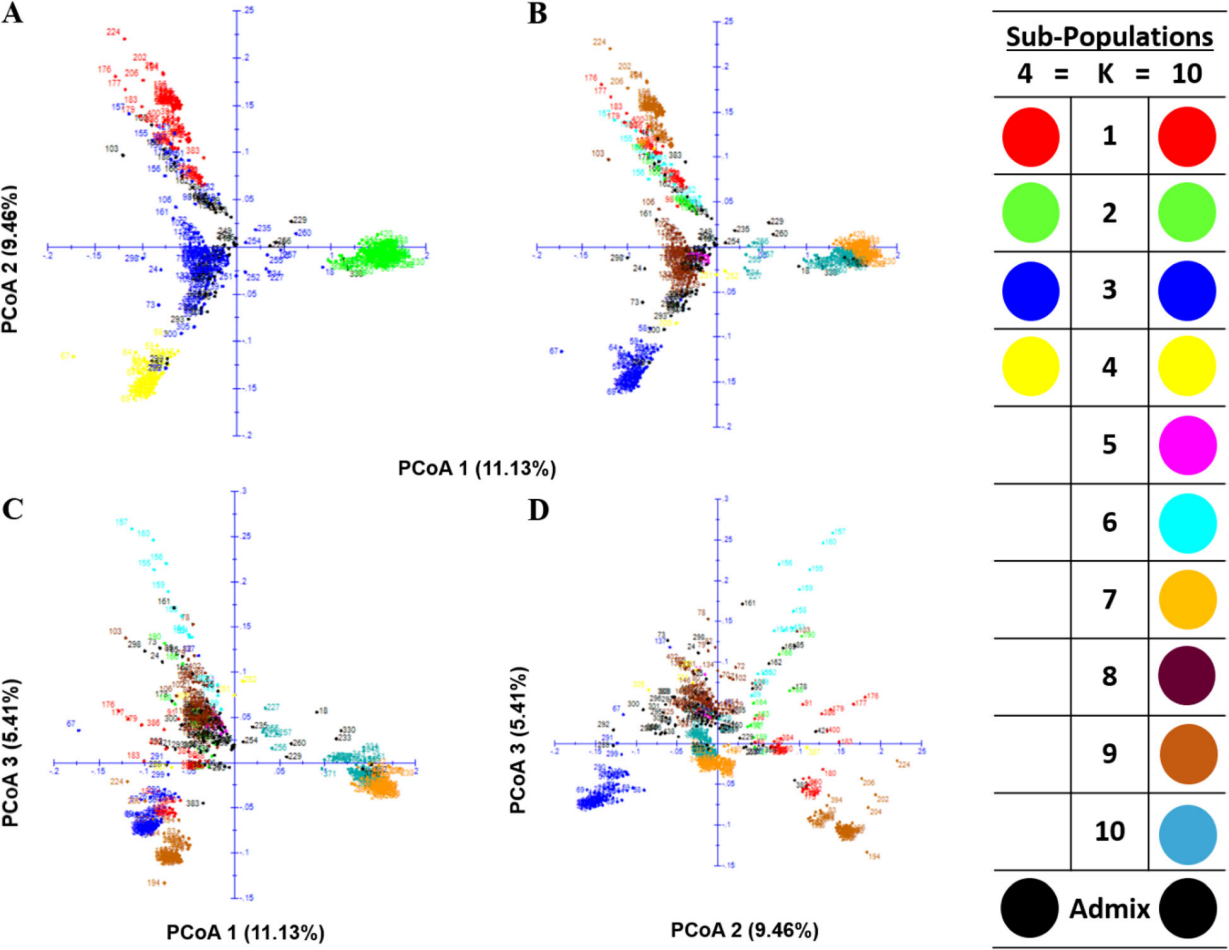
Principal coordinate analysis (PCoA) of the 439 maize inbred lines. Colour-coded according to membership (based on > 60% identity) to sub-populations identified from structure analysis at K = 4 (**a**) and 10 (**c**-**d**)

The Neighbor-joining (NJ) method assigned all the 439 inbred lines into four clusters (C_1_ to C_4_) which were further re-grouped into two main-clusters (A and B) (Fig. 3). For the purpose of comparison, each branch of the tree was displayed with the same colour as in the STRUCTURE analysis with K = 4 and K = 10 and the respective sub-population denoted by roman numerals (I to IV) and with numerical digits (1 to 10), respectively (Fig. 3a and b). Broadly, the groupings of the inbred lines based on the PCoA were also in accordance with the NJ-clustering and model-based population partition in grouping lines into the different sub-populations (Figs. 3 and 4). The PCoA explained 20.59% of the total SNP variation among inbreds across the first two axes. The two-dimensional scatter plot showed that PCoA 1 and PCoA 2 accounted for 11.30 and 9.46% of the total variation, respectively, revealing the presence of four major groups (Fig. 4a).

Despite the inconsistency in the NJ-clustering and STRUCTURE analysis at K = 4 and 10 (Fig. 3), the PCoA clearly differentiated the sub-population-I (SP-I; red colour; K = 4; comprising 76 inbreds) corresponding to cluster C_1_ into two groups (1 and 9) and supported the population structure of the panel of inbred lines obtained at K = 10 (Fig. 4). Furthermore, the PCoA indicated substantial differences in the level of intra-population structure in groups 1 and 9 (Fig. 4d). The STRUCTURE analysis at K = 10 showed group 1 as comprising 6.83% (red; composed of 30 inbreds) of the panel of inbred lines containing both yellow, orange and white endosperm kernel lines derived from various germplasm sources including TZEE-Y Pop STR 106 and 2009 TZE OR1 DT STR (having *Zea diploperennis* background), as well as inbreds extracted from the normal white endosperm germplasm sources such as TZEE-W Pop STR, TZEE-W Pop x LD S6 and TZE-W Pop STR. Six testers comprising early maturing orange (TZEIOR 108), and extra-early maturing yellow (TZdEEI 7 and TZdEEI 12) as well as white endosperm kernel testers (TZdEEI 50, TZEEI 13 and TZEEI 21) were also classified with inbred lines in group 1 (Additional file 2: Table S1). Forty-eight inbred lines constituting group 9 (dark brown colour) represented 10.93% of the panel of inbreds extracted from either the orange/yellow kernel endosperm, broad based populations TZEE-Y Pop Co, TZEE-Y SR × 1368 STR, 2009 TZE OR1 DT STR and TZEE-Y Pop STR 106, or the biparental population (TZEI 17 x TZEI 11). The only exception was the inbred TZdEEI 71 developed from the population, TZE-W Pop STR 107 (Figs. 3 and 4; Additional file 2: Table S1). Furthermore, an early maturing orange kernel endosperm inbred tester, TZEIOR 129 and two extra-early yellow endosperm inbred testers, TZEEI 79 and 81 were also placed in group 9.

The first coordinate axis (PCoA1) described genetic differentiation between sub-population II (SP-II; green colour; K = 4; 111 inbred lines) corresponding to C_2_ (NJ clustering) and the other clusters. Furthermore, the STRUCTURE analysis at K = 10 suggested that SP-II comprised group 7 (orange colour; consisting of 71 inbred lines) and group 10 (oak colour; consisting of 37 lines) representing 16.2 and 9.8% of the panel of 436 inbred lines, respectively. However, both groups were not well separated by the first three coordinates of the PCoA indicating their proximity at the genetic level (Fig. 4). Group 7 consisted of both white and orange/yellow kernel inbred lines derived from varying genetic backgrounds such as the white endosperm kernel bi-parental cross TZEI 1 x TZEI 2, yellow endosperm kernel bi-parental crosses TZEI 17 x TZEI 11 and TZEI 11 x TZEI 8, the broad based orange/yellow endosperm populations, TZE Comp5-Y and 2009 TZEE OR1 STR and the broad-based white endosperm kernel populations TZEE-W Pop Co, WEC STR, TZE-W Pop x LD, TZE-W Pop × 1368 STR and TZE-W Pop STR Co. Two extra-early maturing orange kernel inbred testers, TZEEIOR 109 and TZEEIOR 197 derived from 2009 TZEE OR1 STR also belonged to group 7. Group 10 consisted of only yellow kernel inbred lines extracted mainly from the bi-parental population, TZEI 11 x TZEI 8 together with some few inbred lines including the yellow endosperm tester TZEI 23 extracted from the broad-based population, TZE-Y Pop STR (Additional file 2: Table S1).

The C_3_ (NJ-cluster) contained the highest number of inbred lines and consisted of sub-populations SP-III and SP-IV, whereas C_4_ having the lowest number of inbred lines constituted most of the admixture lines together with few inbred lines representing SP-III. This revealed the inconsistency in the results of the NJ- cluster and STRUCTURE analyses when considering the K value of 4 (Fig. 3a). High level of similarity was observed in the clustering patterns of STRUCTURE (K = 4 and K = 10) and PCoA for SP-IV/group 3 (Figs. 3 and 4). This sub-population consisted of 15.3% of the panel of inbred lines extracted from TZEI 1 x TZEI 2, TZEE-W Pop STR 108, TZE-W Pop STR 108 and TZEE-W Pop STR 104, including an early maturing white endosperm kernel tester (TZdEI 100) developed from TZE-W Pop STR 104 (Additional file 2: Table S1). Similarly, some inbred lines representing SP-III (blue colour) but grouped with members of SP-1 in C_1_ were also clearly differentiated by PCoA, further supporting the new group 6 revealed by STRUCTURE analysis at K = 10 (Fig. 4). All the inbred lines in group 6 (Silver oak, 13 inbreds constituting 2.96% of the panel of inbred lines) contained orange endosperm kernels and originated from 2009 TZE OR1 DT STR population except inbred TZdEEI 13 with low threshold (0.6), derived from TZEE-Y Pop STR*.*

Interestingly, some orange endosperm kernel inbred lines in cluster 3 (C_3_) classified as admixture by the STRUCTURE analysis at K = 4 formed new group 2 when the value of K was considered as 10 (Additional file 2: Table S1). Nonetheless, PCoA clearly differentiated the group 2 (Fig. 4b-d; green colour) but showed their proximity with group 6 suggesting that these groups were very similar. The group 2 representing 2.05% of the panel of inbred lines also shared their pedigree with group 6 which had several inbred lines derived from 2009 TZE OR1 DT STR, a broad-based orange endosperm kernel, drought tolerant and *Striga* resistant population. Although, NJ clustering partitioned the SP-III (blue colour) of the inbred lines panel at K = 4 into three clusters including some inbreds in C_1_ with SP-I (Fig. 3a), the lines were not well separated by PCoA into different groups (4, 5 and 8) except the lines in group 6 which were clearly separated by STRUCTURE analysis at K = 10 (Fig. 4b-d). The group 8 (brown colour, consisting of 75 inbred lines) constituted the highest proportion of the panel of inbred lines (17.08%) and were extracted from the broad-based populations, particularly from the broad-based early orange population 2009 TZE OR1 DT STR as well as the extra-early orange population (2009 TZEE OR1 STR) and the broad-based white endosperm early (TZE-W Pop STR 105 and TZE-W Pop STR 107) and extra-early white endosperm populations (TZEE-W Pop STR 104 and TZEE-W Pop STR 105). Three testers including both extra-early orange (TZEEIOR 30 and TZEEIOR 250) and early white endosperm inbred line, TZdEI 352 possessing *Striga hermonthica* resistance, low-N and drought tolerance and derived from *Zea diploperennis* also corresponded to group 8. It is interesting that all the inbred lines of group 5 (pink colour) representing approximately 2% of the panel of the inbred lines had *Zea diploperennis* background and originated from the broad-based *Striga* resistant yellow early (TZE-Y Pop STR) as well as extra-early (TZEE-Y Pop STR) populations whereas group 4 (yellow, comprising < 1.0% of the panel of inbred lines) contained only four inbred lines extracted from diverse genetic backgrounds (Additional file 2: Table S1). None of the testers were placed in groups 2, 5 and 6 while five testers (ENT 13, TZEEI 29, TZEEIOR 30, TZEI 10 and TZEI 17) had less than 60% probability of association, and hence were classified as admixture (Additional file 2: Table S1).

## Discussion

Manifestation of heterosis and its fixation remain the preferred choice for maximizing gains from selection in crop plants and largely depends on the level of genetic diversity of germplasm base. The advent of PCR based markers, greater genome abundance and high reproducibility, have made SSR markers the ‘marker of choice’ but the availability of high-density genotyping technologies have resulted in a shift from SSR makers to SNP markers such as DArT which are amenable to high-throughput technology and are considered as ‘marker in demand’ [18, 37]. In the recent past, DArTseq marker platforms have been successfully used to quantify diversity in cereals including maize [36, 38–44]. The mean PIC value for the SNP dataset in the present study was 0.206 (ranging from 0.029–0.375) and was comparable with the PIC value estimated for tropical maize by Adu et al. [36], both in terms of mean value (0.19) and range (0.01–0.38) but lower than those described by Wu et al. [44] and Zhang et al. [45]. In previous studies, low PIC value for IITA maize germplasm has also been reported when compared with temperate, INERA and CIMMYT germplasms [46]. The low to moderate genetic diversity observed in the IITA maize germplasm may be attributed to the breeding strategies adopted at IITA which cut across the extra-early, early, intermediate, and late maturing groups [47]. The maize inbred panel used in our study consisted of 439 early and extra-early maize inbreds, which was a good representation of the genetic variation of contemporary IITA early and extra-early maturing maize germplasm. Previous diversity studies of early and extra-early maturing tropical maize involved much fewer inbred lines: 17, 22, 92 and 94 have been reported by Badu-Apraku et al. [48], Akaogu et al. [49], Ifie [35] and Adu et al. [36], respectively.

The population structure is important for explaining the heterogeneity of genetic architecture and is mainly affected by spatial and gene exchange isolation [50]. Based on 9642 DArT markers, population structure and patterns of relationship of 439 inbred lines was investigated based on different complementary approaches that clearly revealed the existence of genetically distinct groups in the present panel of inbred lines (Figs. 3 and 4). Our results revealed that the pattern of grouping from population STRUCTURE analysis and PCoA methods was more reliable than the Neighbor-Joining clustering method. These findings are consistent with those reported by Semagn, et al. [30]. Nonetheless, the agreement between STRUCTURE and PCoA methods was unexpected, as PCoA summarized variations between pre-defined groups based on population structure. Contrarily, NJ-cluster showed low concordance with STRUCTURE analysis in respect of the number of groups and assignment of genotypes to their respective groups (Fig. 3). However, clustering methods are prone to possible ambiguity, since a single distance matrix and a clustering algorithm may give rise to several other clusters [30, 46, 51]. The similarity in grouping patterns obtained with PCoA suggested that the groupings obtained were reasonably reliable despite the discrepancies in number and size of sub-populations/groups (Fig. 4).

Since the late 1990s, when there was a major shift in emphasis from maize breeding for open-pollinated varieties towards hybrid development in WCA region, several efforts have been made to classify the numerous IITA early and extra-early maize inbred lines into heterotic groups using different methods including phenotypic data of measured traits, combining ability effects of multiple traits and molecular markers, but heterotic groups are still not fully established [15, 47, 52, 53]. Akinwale et al. [47] suggested four to five heterotic groups on the basis of the combining ability analysis of selected early white and yellow maize inbred lines and concluded that grouping of inbreds using information from only combining ability studies could lead to contradictory results due to G x E interactions and could result in the classification of the same inbred lines into different heterotic groups in different studies as it relied largely on yield which is a polygenic trait with high influence of environment.

In the present study, different multivariate methods were used to group the panel of IITA-MIP early and extra-early inbred lines into four major clusters, but close examination of the available information clearly indicated greater number of sub-populations. Our results revealed clear population stratification which was consistent with the ancestry, selection history and kernel colours of the inbred lines (Figs. 3 and 4; Additional file 3: Table S2). For example, NJ-clustering, STRUCTURE analysis and PCoA methods consistently grouped all the inbred lines extracted from two early broad-based populations (TZE-W Pop STR 108 and TZE-W Pop STR 104) into a single group (SP-IV and sub-population 3 at K = 4 and 10, respectively) along with lines from other pedigree sources (TZEE-W Pop STR 108, TZEI 1 x TZEI 2 and TZEE-W Pop STR 104) with white endosperm kernels and *Striga* resistant characteristics (Table 2; Figs. 3 and 4). The early maturing population TZE-W Pop was formed by recombining Pool 16 DT, Pool 16 sequoia C_2_, DR-W Pool BC_1_F_1_ and an intermediate maturing inbred 5012 while TZEE-W Pop is an extra-early population derived from recombination of diallel crosses among the outstanding extra-early white varieties, Pool 27 × Gua 314 BC_1_, Pop 30 × Gua 314 BC_1_, TZEE-W SR × Gua 314 BC_1_and TZEE-W SR BC_5_ [54]. The inbreds TZEI 1 and TZEI 2 also contained germplasm of TZE-W Pop background with improved *Striga* resistance. The grouping of inbreds extracted from TZE-W Pop STR and TZEE-W Pop STR was expected because *Striga* resistance trait was incorporated into these populations from the *Striga* resistant intermediate maturing inbred TZi 3 (1368 STR) [55, 56]. Furthermore, the inbred lines in five groups (2, 5, 6, 9 and 10) had yellow/orange kernels while the remaining groups (1, 4, 7 and 8) contained both white and yellow/orange endosperm inbred lines (Additional file 2: Table S1). All the inbred lines including some testers containing genes from *Zea diploperennis* background were clustered into five groups (1, 3, 5, 8 and 9). It was striking that all the inbred lines of group 2 and 6 were derived from a common source, 2009 TZE OR1 DT STR while other groups contained inbreds from different pedigree sources suggesting the existence of substantial diversity within the population or pool from which the inbred lines were extracted [35, 51]. For example, clustering of inbreds derived from the orange/yellow endosperm broad-based population (2009 TZE OR1 DT) and the bi-parental population (TZEI 17 x TZEI 11) in group 9 and most of the inbred lines from the yellow endosperm broad-based population (TZE-Y Pop STR Co) and bi-parental population (TZEI 11 x TZEI 8) in group 10 indicated some common attributes in their ancestry (Fig. 3 B and Fig. 4; Additional file 2: Table S1). It is noteworthy that these inbreds were extracted from TZE-Y Pop DT STR and TZE Comp5-Y DT populations improved for drought tolerance and have DR-Y Pool BC_2_F_2_, KU 1414, and TZi 28 (9499) in their genetic backgrounds as the sources of drought tolerance [57, 58]. The TZEE-Y Pop is an extra-early yellow endosperm broad-based population formed by compositing CSP-SR BC5, TZEE-Y SR BC5, CSP × Local Raytiri, and TZEE-Y populations while TZE-Y Pop STR is an early yellow endosperm broad-based population with resistance to *Striga* and tolerance to drought and was developed by recombining DR-Y Pool BC2F2, KU1414 and the intermediate maturing yellow endosperm inbred line 9499 [57]. Similarly, TZE-Comp 5 is an early maturing population derived by crossing TZESR-WC3 to 10 *Striga* resistant inbred lines [59]. Therefore, the lack of clear heterotic patterns in tropical maize germplasm compared to temperate germplasm is mainly attributed to the earlier maize breeding focus on the development of broad-based populations and genetic pools at both CIMMYT and IITA [16, 33]. This might further explain the reason for the low to moderate diversity in the IITA early maturing maize germplasm, as selection pressure was directed more towards fixing of the favourable allele frequency for specific characteristics such as maturity period (early to extra-early), biotic (MSV and resistance to *Striga*) and abiotic (tolerance to drought) stresses in the populations via recurrent selection. Thus, the complex clustering patterns of the present set of maize inbred lines was not unexpected as the mixed genetic constitution of the populations and pools may be due to the grouping together of inbreds derived from different base populations. Nevertheless, this has made the task of assigning inbreds into distinct heterotic groups at molecular level difficult. This corroborates the findings of earlier researchers in which molecular markers displayed the existence of complex population structure in tropical maize, including CIMMYT maize lines (CMLs) and researchers were unable to group them into complementary heterotic patterns [30, 33, 46, 51].

**Table 2:** Details of source populations of 439 early and extra-early maturing maize inbred lines used in the present study

SN	Source population	Number of inbreds	Grain colour	Origin
1	[M37W/ZM607#bF37sr-2-3 sr-6-2-X]-8–2-X-1-BB-B-xP84c1 F27–4–3-3-B-1-B] F29–1–2-2 x [KILIMA ST94A]-30/MSV-03–101-08-B-B-1xP84c1 F27–4–1-4-B-3-B] F2–1–2-1-1-1-B x CML486]-1–1	1	yellow	CIMMYT
2	[(87,036/87923)-X-800-3-1-X-1-B-B-1-1-1-B-B-xP84c1 F26–2–2-4-B-2-B] F47–3–1-1-3 x M37W/ZM607#bF37sr-2-3 sr-6-2-X]-8–2-X-1-BB-B-xP84c1 F27–4–3-3-B-1-B]-3–2-B x P33c3 F64–1–1-4-BB]-1–1	1	yellow	CIMMYT
3	[M37W/ZM607#bF37sr-2-3 sr-6-2-X]-8–2-X-1-BB-B-xP84c1 F27–4–3-3-B-1-B] F29–1–2-1-4 x (87,036/87923)-X-800-3-1-X-1-B-B-1-1-1-B-B-xP84c1 F26–2–2-4-B-2-B]-1–1-B x CML486]-1–1	1	yellow	CIMMYT
4	TZEE-Y Pop STR 106	16	yellow	IITA
5	TZE-Y Pop STR 106	8	yellow	IITA
6	TZE-W Pop STR 104	15	white	IITA
7	TZEE-W Pop STR 104	6	white	IITA
8	TZE-W Pop STR 105	3	white	IITA
9	TZEE-W Pop STR 105	5	white	IITA
10	TZE-W Pop STR 107	11	white	IITA
11	TZEE-W Pop STR 107	2	white	IITA
12	TZE-W Pop STR 108	24	white	IITA
13	TZEE-W Pop STR 108	8	white	IITA
14	2009 TZE OR1 DT STR	111	orange	IITA
15	TZE Comp5-Y C6	6	yellow	IITA
16	TZE-W Pop × 1368 STR	4	white	IITA
17	TZE-W Pop x LD	1	white	IITA
18	WEC STR	2	White	IITA
19	TZE-Y Pop STR Co	16	yellow	IITA
20	TZE-W Pop STR Co	6	white	IITA
21	(TZEI 1 x TZEI 2)	66	white	IITA
22	(TZEI 11 x TZEI 8)	49	yellow	IITA
23	(TZEI 17 X TZEI 11)	20	yellow	IITA
24	TZEE-W Pop x LD	8	white	IITA
25	TZEE-W SR BC5 × 1368 STR	3	white	IITA
26	TZEE-Y SR BC1 × 9450 STR	5	yellow	IITA
27	TZEE-Y Pop Co	1	yellow	IITA
28	TZEE-W Pop Co	1	white	IITA
29	2009 TZEE-ORI STR	37	orange	IITA
30	TZEE-W Pop × 1368 STR S7 Inb 40 x Pool 15 SR QPM	2	white	IITA
Total		439		

Knowledge of the genetic relationship among testers and their efficiencies in grouping other inbred lines is important for a hybrid breeding program to be successful. Therefore, plant breeders are continuously studying inbred testers to determine their efficiencies in grouping other inbred lines. Several promising testers have been identified in the IITA early and extra-early maize improvement program over the past twenty years, but precise information with respect to their specific heterotic groups is still not fully established [16]. In agreement with earlier reports, the two inbred testers, TZdEEI 12 and TZdEEI 7 belonging to the same heterotic group were classified into group 1 while TZEEIOR 109 and TZEEIOR 197 assigned to group 7 also belong to similar heterotic group (Additional files 2 and 4: Tables S1 and S3).

Based on the results of the present study, IITA-MIP breeders could formulate breeding strategies for genetic improvement of early and extra-early maize in SSA. Planned crosses involving representative testers from opposing heterotic groups identified in the present study could be initiated to refine the existing heterotic groups in the IITA-MIP. Results presented in this study could serve as an important guide to parent selection for further population improvement and development of productive hybrids in the IITA-MIP to maximize maize productivity in different agro-ecologies of SSA region. For example, the classification of the maize inbreds into distinct heterotic groups in the present study is expected to facilitate the development of superior hybrids, synthetics, pools and breeding populations possessing resistance/tolerance to multiple stresses (such as drought, low-N, and *Striga hermonthica*) as well as enhanced nutritional qualities including PVA and quality protein levels of tropical maize. Additionally, the information obtained from the DArT-SNP marker-based genetic distance (GD) estimates can employed to minimize the cost of testing in the IITA-MIP by preventing evaluation of crosses between related lines and assist in eliminating crosses with poor performance [60]. Furthermore, the results of the molecular analyses could be combined with morphological and agronomic testing of the IITA-MIP germplasm to provide complementary information and increase the resolving power of genetic diversity analyses [61]. Finally, the identification of diverse parental combinations will facilitate the creation of segregating progenies with maximum genetic variability for further selection [62] and the introgression of favourable alleles from diverse germplasm sources into available breeding populations as proposed by Thompson et al. [63].

The strategy of IITA-MIP has been to establish a pair of heterotic groups each for the different maturity classes, based on the kernel colour and target breeding objectives using line x tester mating design, North Carolina Design II (NCD II), Diallel mating design, and grouping methods such as SCA effects of grain yield, heterotic grouping based on general and specific combining ability effects of grain yield (HSGCA), heterotic grouping based on general combining ability effects of multiple traits (HGCAMT) and DNA markers. Presently, a pair of heterotic groups has been established in the IITA-MIP for developing white normal endosperm hybrids as well as white QPM hybrids of early and extra-early maturity classes (Additional file 5: Figure S2). Similarly, we have a pair of heterotic groups targeted at developing yellow and orange normal endosperm as well as yellow QPM, and orange QPM hybrids of early and extra-early maturity classes. In practice, it is ideal to have two heterotic groups for each maturity class and endosperm colour for a successful practical maize breeding program. Therefore, the four heterotic groups identified in the present study could pose a major challenge to the present strategic decision of the IITA-MIP to classify the inbred lines in the program into a maximum of three heterotic groups designated as A, B, and C (the mixed group). The number and choice of heterotic groups are arbitrary decisions and a breeding program can have two or more heterotic groups. However, working with two distinct heterotic groups, designated as A and B with subgroups within each group for different maturity classes and endosperm colors would facilitate the management of the heterotic groups and accelerate genetic gains from selection. Nevertheless, several challenges would need to be addressed if this strategy is adopted in our program to ensure accurate classification of invaluable inbred lines in the mixed group C that falls outside the classical A and B heterotic groups. Therefore, our goal is to reduce the heterotic groups identified in the present study into A and B categories. This could be achieved by aligning the heterotic affinities of the elite inbred lines with mixed genetic backgrounds into existing heterotic groups A and B using field evaluations of crosses with testers of known heterotic groups and molecular markers. The heterotic groups of some of the inbred lines derived from the breeding populations in the present study which have been used in developing commercial hybrids in the IITA-MIP are presented in the Additional file 6: Table S4 and Additional file 7: Figure S3. The inbred lines have been classified into heterotic groups A and B. In an effort to determine whether the classification of the inbred lines into heterotic groups based on SNP markers was reasonably reliable, the selected inbred lines which have been used in developing commercial hybrids in the IITA-MIP were grouped using SNP markers in the present study. The groupings based on the SNP markers were then compared with those based on the mating designs and grouping methods such as the SCA of grain yield, HSGCA and HGCAMT. The classification of the selected early white, yellow, and orange endosperm inbred lines into heterotic groups A and B using SNP markers approximated 64 and 56% respectively for the lines that should have been classified based on the different multivariate methods. Similarly, placement of the extra-early white, yellow and orange endosperm inbred lines into heterotic groups A and B using SNP markers approximated 71 and 50%, respectively compared to the groupings based on the different multivariate methods. The results of this study revealed close correspondence between the groupings of the inbred lines based on the mating designs/classical grouping methods and the SNP markers. However, there is a need for continuous refinement of the heterotic groups to ensure continuous and adequate genetic gains from selection in the IITA-MIP extra-early and early breeding program. Finally, it should be noted that it would be impracticable to have as many as 24 heterotic populations for the early and extra-early maturity groups alone as presented in Additional file 5: Figure S2, so a strategy has to be developed to prioritize the number of heterotic groups that would be manageable and cost effective for the IITA-MIP extra-early and early breeding program.

## Conclusions

The present study has provided useful information on the genetic variability and population structure of early and extra-early maize inbreds with wide adaptation to the different agro-ecologies of the SSA. Using DArTseq technology, the multivariate methods identified four distinct groups which are generally in agreement with the ancestry, selection history and kernel colour of the lines but indicated a complex pattern of genetic structure. Our results suggest that the application of complementary approaches is very efficient in predicting the presence of groups and in placing the genotypes into the different groups based on molecular markers. As an additional tool, the molecular markers are useful for preliminary assignment of inbred lines into prospective groups where discrete heterotic groups are not well established. Nonetheless, the grouping of testers into each potential heterotic group may help reduce the number of actual field crosses that would need to be made to validate the grouping of these inbred lines with a limited number of field evaluations of the crosses. Finally, our study has demonstrated the existence of high level of diversity among the present set of early and extra-early inbred lines of IITA with good adaptation to the SSA maize growing conditions in countries including Nigeria, Ghana and Mali. Consequently, during the past decade, molecular approaches have been adopted in the IITA-MIP to refine genetic diversity and combining ability studies and this has resulted in increased hybrid maize productivity at relatively faster and cheaper rates.

## Methods

### Plant materials

Four hundred and thirty-nine diverse maize inbreds widely adapted to agro-climatic conditions in SSA were used in the present study (Additional file 3: Table S2). This germplasm comprised 436 inbreds (342 early and 94 extra-early) and three inbreds developed by IITA and CIMMYT maize breeding programs, respectively. These inbreds were developed from twenty-seven broad-based and three narrow-based source populations derived from both exotic and local germplasm sources identified based on several years of multilocation evaluations for adaptation to the different agro-ecologies of SSA region (Table 2). Some of the inbred lines in the panel represent sources of several outstanding multiple stress resistant/tolerant early and extra-early maturing commercial maize OPVs and hybrids released in different WCA countries. For instance, an extra-early maize hybrid (Ife-Maizehyb5), and four early maturing hybrids (Sammaz 41, Sammaz 42, Sammaz 46 and Sammaz 47) released in Nigeria; seven hybrids comprising four extra-early (Obotantim, Nkabom, CSIR-Komnaaya and CSIR-Wang-Basig) and three early (Kunjor-wari, CSIR-Similenu and CSIR-Denbea) maturing hybrids released in Ghana; four early maturing commercial hybrids designated as Dilika, Sanu, Apraku and Tamalaka released in Mali. Moreover, the panel also contains some commonly used testers in IITA-MIP such as the extra-early-maturing white QPM inbred (TZEEQI 7), early-yellow inbred testers (ENT 13, TZEI 10, TZEI 17, TZEI 23), early maturing orange inbred testers (TZEIOR 25, TZEI 124, TZEIOR 108 and TZEI 129) and early white inbred testers (TZEI 100, TZEI 7, TZEI 18, TZdEI 352 TZEI 19 and TZdEI 100) and extra-early maturing orange testers (TZdEEI 7, TZdEEI 12, TZEEIOR 30, TZEEIOR 97 and TZEEIOR 197), extra-early yellow inbred testers (TZEEI 79, and TZEEI 81) and extra-early white inbred testers (TZdEEI 50, TZEEI 21, TZEEI 13 and TZEEI 29).

### Sample preparation and DNA isolation

For genomic DNA extraction, leaf samples from 8 to 10 seedlings of each inbred line were collected at 3 weeks after planting and stored in the deep freezer (− 80 °C), freeze-dried and ground as described by Adu et al. [36]. Total genomic DNA from each sample was extracted following standard DArT procedure [36]. In a 96 well plate, ninety-four samples were placed and individual plates were sealed in accordance with DArT instructions. Finally, all the plates were kept in a shipping box and dispatched to the DArT P/L platform, Genetic Analysis Service for Agriculture (SAGA) facility at CIMMYT, Mexico.

### DArTseq genotyping, data filtering and statistical analysis

Wide-genome genotyping of the 439 inbred lines was conducted using DArTseq technology [21, 40]. Following a strict quality control process involving parameters such as call rate, data reproducibility (~ 20% of samples replicated), and rate of monomorphism to remove monomorphic markers, 18,927 SNPs were obtained from the studied germplasm. Molecular markers were filtered again utilizing PLINK 1.9 software and those showing greater than 20% missing data were removed. Moreover, SNPs having a variance close to 0 and the rare SNPs with less than 5% minor allele frequencies (MAF) were also eliminated from the dataset resulting in final dataset containing 9642 DArTseq informative SNPs.

Statistical analysis for genetic diversity parameters including MAF, unbiased estimation of gene diversity, observed and expected heterozygosity (H_o_ and H_e_), and PIC value were performed using PowerMarker V3.25 software [64].

### Genetic structure analysis

To reveal the genetic structure of the panel of maize inbred lines, all the 9642 DArTseq markers were imported into the Bayesian Markov chain Monte Carlo software STRUCTURE V2.3.4 [65]. In the ADMIXTURE method, the number of sub-populations varying from k = 1–20, and five times simulations with iterations and burn-ins set to 10,000 were used, with no prior information on the origin of individuals [19]. For the most appropriate k-value within the present panel, the Evanno transformation method was used which is useful and better described the data and also exhibited a low cross-validation error compared to other k values [66]. Following the Evanno ΔK method, the results obtained from STRUCTURE were implemented in Structure Harvester to determine the most suitable value of k. Inbred lines with membership probabilities ≥0.60 were assigned to the corresponding sub-population while less than 0.60 were considered as admixture.

To confirm the assignment of inbreds into the sub-population by STRUCTURE analysis, population phylogeny was also studied by imputing the full set of data into DARwin software [58, 67] using neighbor-joining (NJ) tree feature by running 30,000 bootstraps. The phylogenetic tree was constructed in FigTree version 1.4.3 software [68]. The inbred lines in each cluster of the NJ phylogenetic tree were highlighted by different colours corresponding to the results obtained by the STRUCTURE analysis. Finally, principal coordinate analysis (PCoA) was also carried out utilizing the DARwin software [69] to visualize the pattern of genetic differentiation within and between the groups of inbred lines and complemented the pattern of diversity and clustering revealed by STRUCTURE analysis and dendrogram, respectively.

## Supplementary Information

**Additional file 1: Figure S1.** Member probability of the inbred lines at k = 4 and k = 10 using different assignment thresholds (60, 70, 80 and 90%).

**Additional file 2: Table S1.** Assignment of the 439 maize inbred lines into clusters based on a cutting of 60% of threshold.

**Additional file 3: Table S2.** Pedigree of 439 maize inbred lines used in the present study.

**Additional file 4: Table S3.** Details of inbred testers and/or parents of selected early and extra-early maize hybrids released in West Africa (Ghana, Mali and Nigeria) used in the present study.

**Additional file 5: Figure S2.** Proposed strategy for classification of the IITA early and extra-early maize germplasm into heterotic groups A and B.

**Additional file 6: Table S4.** Classification of selected early and extra-early maize inbred lines into heterotic groups A and B.

**Additional file 7: Figure S3.** (A) Classification of selected extra-early maize inbred lines into heterotic groups A and B using molecular markers. (B) Classification of selected early maize inbred lines into heterotic groups A and B using molecular markers.

## Data Availability

The DArTseq datasets used in the present study have been deposited in the CIMMYT Dataverse (https://hdl.handle.net/11529/10548533).

## Abbreviations

PCoA: Principal coordinate analysis
NJ: Neighbor-joining (NJ)
SSA: Sub-Saharan Africa
MAF: Minor allele frequency
DArTseq: Diversity array technology sequencing
IITA: International Institute of Tropical Agriculture
CIMMYT: International Maize and Wheat Improvement Center
WCA: West and Central Africa
CGIAR: Consultative Group for International Agricultural Research
NARIs: National Agricultural Research Institutes
WECAMAN: West and Central Africa Collaborative Maize Research Network
SAFGRAD: Semi-Arid Food Grain Research and Development
MIP: Maize Improvement Program
SNP: Single nucleotide polymorphism
PIC: Polymorphic information content
GD: Gene diversity
