# Supplementary figures and images for: Genetic diversity and population structure of early and extra-early maturing maize germplasm adapted to sub-Saharan Africa
Source: BMC Plant Biol. 2021 Feb 17;21:96. doi: 10.1186/s12870-021-02829-6 (PMC7888073; doi:10.1186/s12870-021-02829-6)

■  $K = 4$  ■  $K = 10$

Unassigned Inbred lines (%)

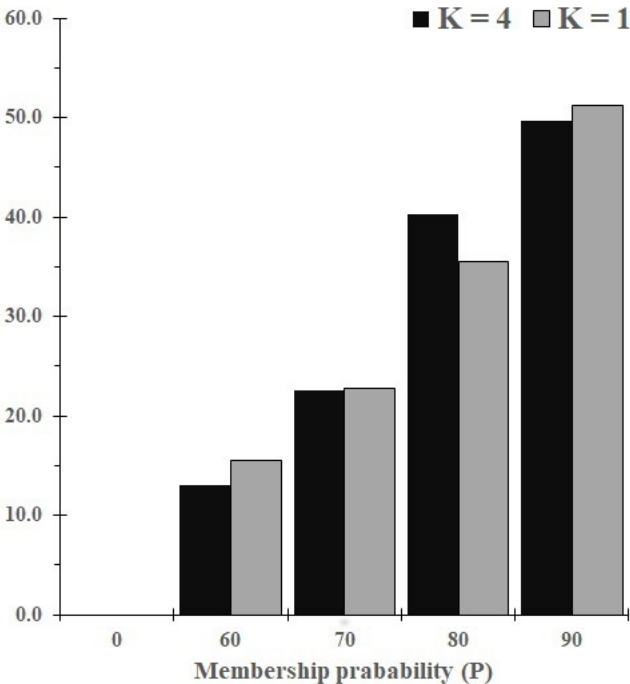

Supplement: Supplementary file 1 — Additional file 1: Figure S1. Member probability of the inbred lines at k = 4 and k = 10 using different assignment thresholds (60, 70, 80 and 90%). [file 12870_2021_2829_MOESM1_ESM.pdf]
