# Supplementary material for: Genetic diversity and population structure of early and extra-early maturing maize germplasm adapted to sub-Saharan Africa
Source: BMC Plant Biol. 2021 Feb 17;21:96. doi: 10.1186/s12870-021-02829-6 (PMC7888073; doi:10.1186/s12870-021-02829-6)
Supplement: Supplementary file 5 — Additional file 5: Figure S2. Proposed strategy for classification of the IITA early and extra-early maize germplasm into heterotic groups A and B. [file 12870_2021_2829_MOESM5_ESM.pdf]

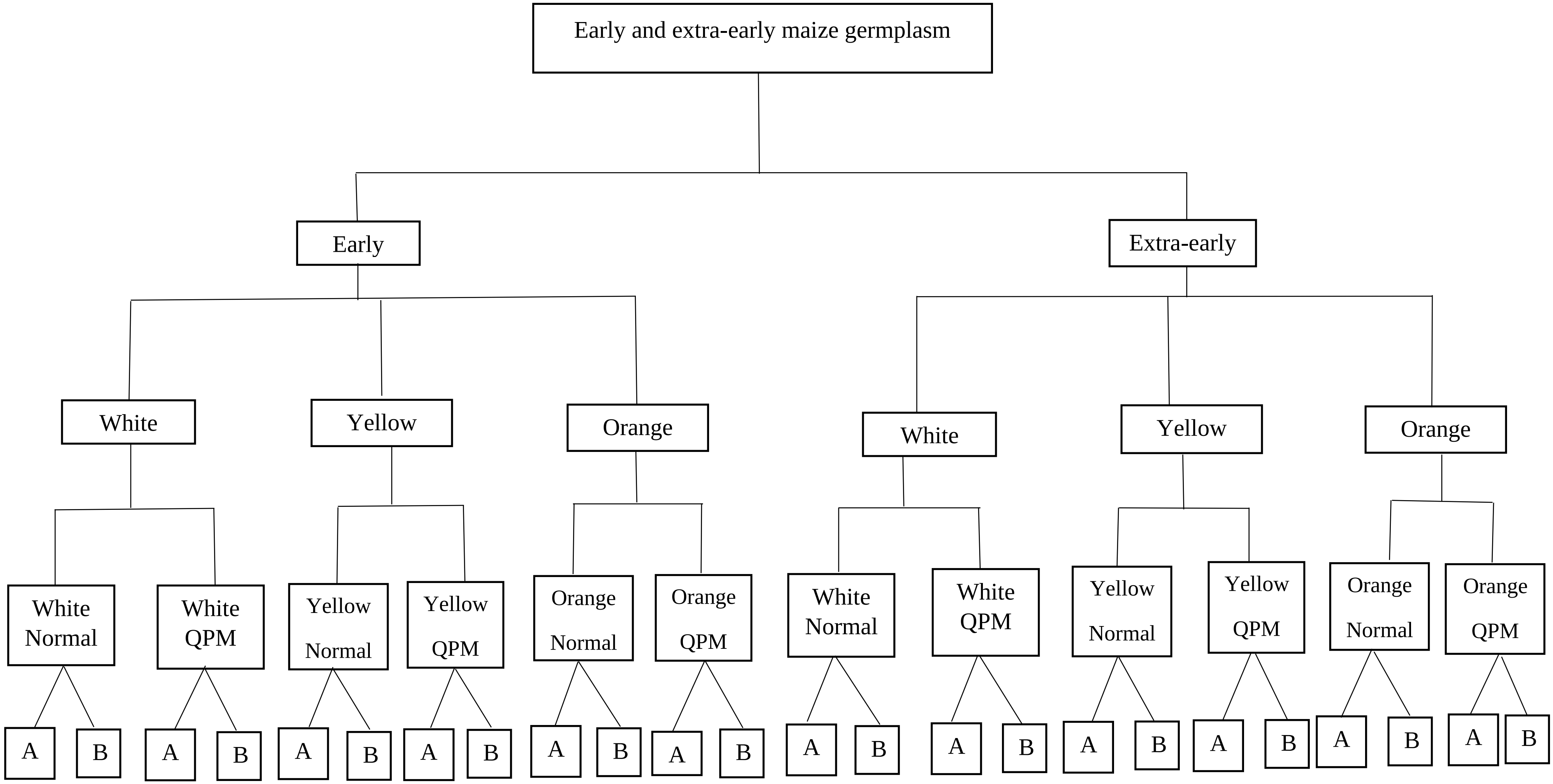

Additional file 5: Figure S2: Proposed strategy for classification of the IITA early and extra-early maize germplasm into heterotic groups A and B.
