# Supplementary material for: Genetic diversity and population structure of early and extra-early maturing maize germplasm adapted to sub-Saharan Africa
Source: BMC Plant Biol. 2021 Feb 17;21:96. doi: 10.1186/s12870-021-02829-6 (PMC7888073; doi:10.1186/s12870-021-02829-6)
Supplement: Supplementary file 7 — Additional file 7: Figure S3. (A) Classification of selected extra-early maize inbred lines into heterotic groups A and B using molecular markers. (B) Classification of selected early maize inbred lines into heterotic groups A and B using molecular markers. [file 12870_2021_2829_MOESM7_ESM.pdf]

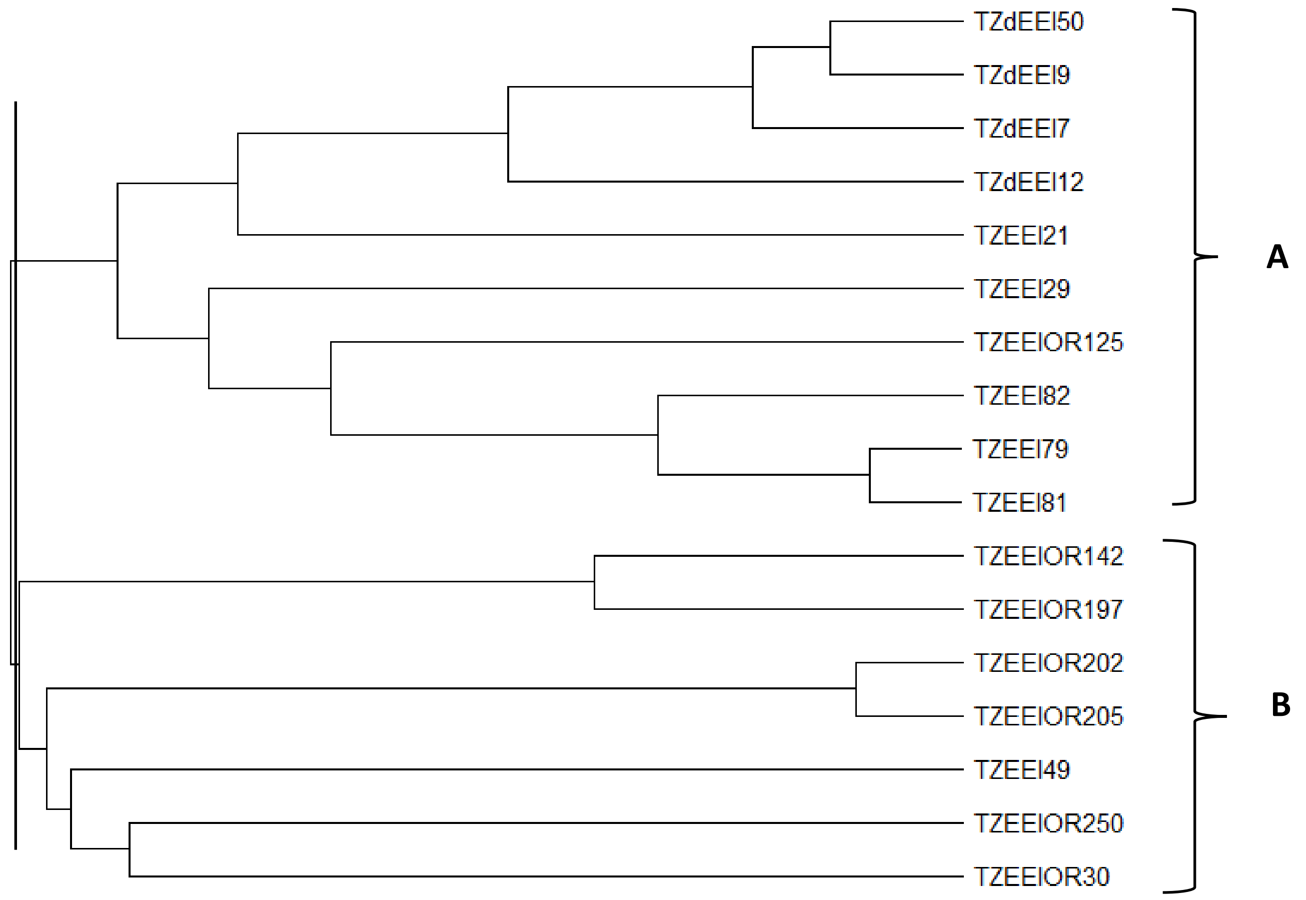

A. Classification of selected extra-early maize inbred lines into heterotic groups A and B using SNP markers.

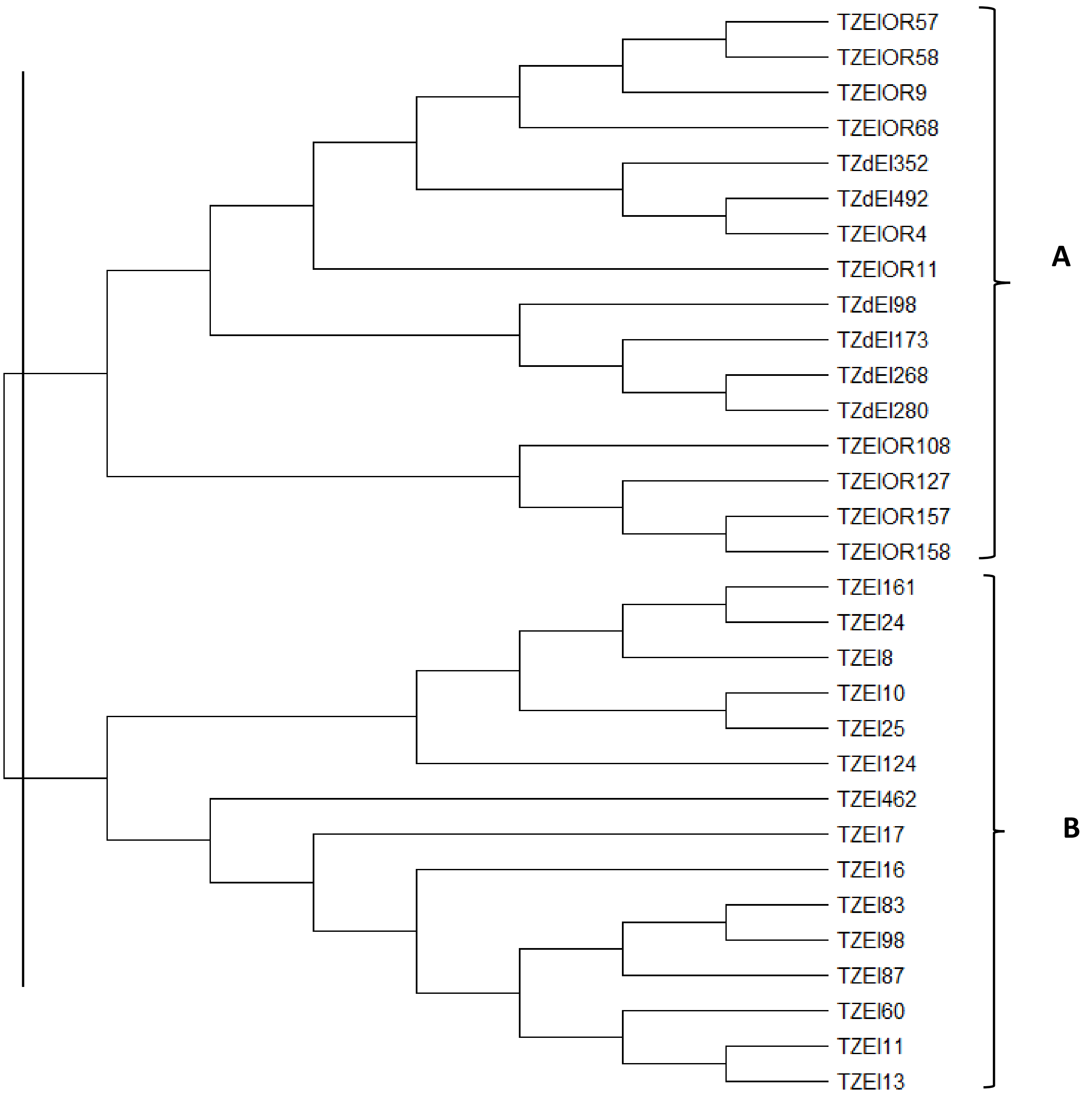

B. Classification of selected early maize inbred lines into heterotic groups A and B using SNP markers.
